# Low levels of the nerve growth factor receptor TrkA in JIA: a possible defect in a novel anti-inflammatory mechanism

**DOI:** 10.1186/1546-0096-9-S1-P102

**Published:** 2011-09-14

**Authors:** G Minnone, R Strippoli, G Prencipe, L De Pasquale, C Bracaglia, A Insalaco, F De Benedetti, L Bracci-Laudiero

**Affiliations:** 1 Division of Rheumatology, Ospedale Pediatrico Bambino Gesù, Rome, Italy; 2 Istituto di Farmacologia Traslazionale-CNR, Rome, Italy

## Background

High levels of Nerve Growth Factor (NGF) are found in chronic inflammatory diseases, including synovial fluids (SF) of juvenile idiopathic arthritis (JIA) patients. The effects of high NGF levels on immune cell activity and inflammatory process are unknown.

## Objective

To evaluate the effects of NGF and the role of its receptor TrkA on cytokine production in monocytes, which express TrkA and are key players of inflammation. To investigate in JIA patients the expression of the NGF receptor, TrkA, in blood and SF mononuclear cells (MC).

## Methods

Using real time PCR we evaluated the expression of TrkA in JIA patients (n=25; mean age 8.93 years) and controls comparable (n=17; mean age 8.93 years). In human monocytes stimulated with LPS, the effect of TrkA activation or inhibition on signalling and cytokine release was evaluated using real-time PCR, western blot and ELISA.

## Results

Compared to controls, TrkA mRNA expression was markedly lower in blood MC of JIA patients (p<0.001). TrkA mRNA levels were similarlv low in SF MC of JIA patients. In vitro, we show that addition of NGF down-regulates IL-6 and IL-1 production and increases IL-10. Blocking the receptor TrkA resulted in enhanced activation of the NF-kB pathway, in increased inflammatory cytokine production and in decreased IL-10.

## Conclusions

We show for the first time that NGF, through TrkA, has anti-inflammatory effects on monocytes, suggesting that the well-known NGF increase found in chronic inflammatory diseases represents an endogenous mechanisms limiting inflammatory response. Defective TrkA expression, as found in JIA patients, may lead to an imbalance between pro-inflammatory and anti-inflammatory pathways contributing to chronic inflammation.

